# Antibiotic resistance and virulence of *Escherichia coli* strains isolated from animal rendering plant

**DOI:** 10.1038/s41598-020-72851-5

**Published:** 2020-10-13

**Authors:** Gabriela Gregova, Vladimir Kmet

**Affiliations:** 1 University of Veterinary Medicine and Pharmacy in Kosice, Komenskeho 73, 041 81 Kosice, Slovakia; 2 Institute of Animal Physiology, Centre of Biosciences, Slovak Academy of Sciences, Soltesovej 4, 040 01 Kosice, Slovakia

**Keywords:** Antimicrobials, Natural hazards, Occupational health, Public health

## Abstract

Processing of animal carcasses and other animal wastes in rendering plants is a significant source of antibiotic resistant microorganisms. The main goal of this study was to investigate the resistance to 18 antibacterial agents including β-lactams, fluoroquinolones, colistin and virulence factors (*iss, tsh, cvaC, iutA, papC, kps* and *ibeA* genes) in 88 *Escherichia coli* strains isolated from a rendering plant over 1 year period. ESBL (*Extended-spectrum beta-lactamases*) and plasmid-mediated Amp were screened by interpretative reading of MIC. ESBL phenotype was detected in 20.4% of samples and high level of resistance to fluoroquinolone was found in 27.2% of strains. Cephalosporinase CTX-M1, cephamycinase CMY-2, integrase 1 and transposon 3 genes were detected by PCR. Furthermore, there were found three CMY-2 producing *E. coli* with O25b-ST131, resistant to the high level of enrofloxacin and containing the gene encoding the ferric aerobactin receptor (*iutA*). One enrofloxacin resistant *E. coli* strain possessed *iss, ibeA, kps* and *papC* virulence genes also with CMY-2, integrase1 and Tn3. ST131 *E. coli* with CMY-2 has a zoonotic potential and presents a serious health risk to humans.

## Introduction

*Escherichia coli* is regarded as a representative indicator of antimicrobial resistance of Gram-negative bacteria.

Risk of transmission of β-lactam resistance to nosocomial *E. coli, es*pecially the increasing resistance to 3rd and 4th generation cephalosporins and carbapenems became a serious issue worldwide^1^.

AmpC β-lactamases are enzymes commonly isolated from *Enterobacteriaceae* and a few other cephalosporin resistant bacteria, encoded on the chromosomes. They mediate resistance to cephalosporins (cefoxitin, cefazolin, cephalothin), most penicillins and β-lactamase inhibitor combinations^2^. Jacoby^2^ described induction or overexpression of chromosomally-located, species-specific AmpC genes and the acquisition of plasmid-encoded AmpC genes (e.g. bla_CMY-like_, bla_ACC-like_, bla_DHA-like_) in *E. coli*.

Resistance of *E. coli* to cephalosporins and fluoroquinolones is of considerable concern, as the prevalence of horizontally acquired resistance mechanisms has increased significantly in the past 20 years. High-level resistance to fluoroquinolones is associated with DNA gyrase and topoisomerase mutations although intermediate resistance or enhancement of clinical resistance is possible by chromosomal efflux pump upregulation and/or plasmid-borne genes encoding target site protection (*qnr*), efflux (*qepA*), or modification of fluoroquinolones by acetylase (*aac(6′)-Ib-cr*)^3^.

*Escherichia coli* sequence type 131 (ST131) is a worldwide pandemic clone responsible for the most part of community-onset antimicrobial-resistant infections. A high prevalence of the clone (∼ 30–60%) has been identified amongst fluoroquinolone-resistant *E. coli*. Moreover, it potentially possesses a variety of β-lactamase genes; they most often include CTX-M family β-lactamases, and, less frequently, TEM, SHV and CMY β-lactamases^4^.

ST131 *E. coli* is widely disseminated within the antibiotic-resistant community and was associated with hospital-onset of *E. coli* infection in the UK. It has been identified among ESBL-producing isolates in seagulls and rats, but in Spain the prevalence of the clone amongst poultry and on pig farms was low^4^.

*The E. coli* ST131 clones cause many multidrug nosocomial infections worldwide (cystitis pyelonephritis, abdominal, soft tissue infections, meningitis, osteoarticular infection, myositis, septic shock and other) and probably can produce biofilms^5^.

Processing of food animal carcasses and secondary raw materials of animal origin in rendering plants is a significant source of microorganisms that contributes to the risk of pollution of the environment. A large body of information is available about antibiotic resistance of microorganisms in municipal wastewater treatment plants that can act as reservoirs and environmental suppliers of antibiotic resistance. They may serve as reservoirs for ESBLs and AmpC producing *Enterobacteriaceae*^6–8^.

However, there are still some gaps in our knowledge about the role of rendering plants in the spread of antibiotic resistance.

The aim of the study was to determine the resistance to 18 antibacterial agents including β-lactams, fluoroquinolones, colistin and virulence factors (*iss, tsh, cvaC, iutA, papC, kps* and *ibeA* genes) in *E. coli* isolated from surface swabs collected in the processing space and from waste water produced by the investigated rendering plant.

## Material and methods

A rendering plant is a processing operation where materials of animal origin are recycled. The following processes are involved: unloading of raw material brought for processing, its sorting, primary processing and sampling, sterilisation, separation of fat and feed meals of animal origin, pressing, processing of feed meals, and processing of animal fat. The operation premises are divided to a section used for common processing of materials of categories I and II and (high risk—meat-bone meal have to be burned at a temperature of 850 °C) a separate section for processing of category III materials (lower risk—meat-bone meal can be used for production of pet granules).

Destruction (crushing of material to 50 mm particles) and sterilisation of animal by-products using the temperature of 133 °C and pressure of 3 bars during 20 min ensure high level of sanitization and limit the risk of spread of microorganisms to the environment. The above mentioned parameters are critical for adequate processing of raw materials entering the rendering plant^9^. In the dryer the solid portion is separated from the liquid one (water) and the dried meat-bone meal is pressed during which process the fat is separated from the meat-bone mash. The technological procedure in the rendering plant includes processing of the wastewater and installation of a biological air washer—the components important for reducing the hygiene-epidemiological risks.

### Sampling

Over 1 year period (10 times) swabbing procedure was used to obtain samples from surfaces in the processing section of the investigated rendering plant and additional samples were collected from raw wastewater in rendering plant. Altogether 88 samples were obtained and examined. Each sample from surface swabs and waste water was inoculated and multiplied in Buffered peptone water (Oxoid, Basingstoke, United Kingdom) and then were sub-cultured on Mac Conkey agar (Oxoid) at 37 °C overnight^10^.

### Identification of *E. coli*

The suspect *E. coli* colonies from McConkey agar were identified by a matrix-assisted laser desorption/ionization (MALDI-TOF) biotyper (Bruker Daltonics, Leipzig, Germany). Bacterial extracts for mass spectrometry measurements were prepared as recommended by the manufacturer of the MS instrument. For MALDI-TOF analysis, one colony was spotted onto a ground steel target (Bruker Daltonik GmbH, Leipzig, Germany) and air dried for 15 min.

Each sample spot was overlaid with 2 μl of matrix solution (saturated solution of α-cyano-4-hydroxy-cinnamic acid in 50% acetonitrile with 2.5% trifluoroacetic acid), and again air dried for 15 min. To identify the relevant microorganisms, the raw spectra obtained for each isolate were imported into a BioTyper software, version 2.0 (Bruker Daltonik GmbH, Leipzig, Germany), and analysed without any user intervention^10^.

### Antibiotic susceptibility

Eighty eight isolates of *E. coli* (one sample-one strain) were analysed for their antibiotic susceptibility and for the presence of ESBLs, pAmpC and for the high level fluoroquinolone resistance.

Minimal inhibitory concentrations (MIC) were determined according to VET01-S2^11^ and EUCAST^12^, by a Miditech system (Bratislava, Slovakia) with interpretative reading of MIC^13^. The antibiotics used in the presented study were as follows: ampicillin (AMP), ampicillin and sulbactam (A + IB), ceftazidime (CAZ), ceftazidime with clavulanic acid (CAC), ceftriaxon (CTR), ceftiofur (CFF), cefquinome (CFQ), ertapenem (ETP), gentamicin (GEN), streptomycin (STM), nalidixic acid (NAL), ciprofloxacin (CIP), enrofloxacin (ENR), chloramphenicol (CMP), florfenicol (FLO), tetracycline (TET), cotrimoxazol (COT), colistin (COL).

Phenotypic confirmation of mechanisms of ESBLs and pAmpC to the β-lactams (CTR, CAZ, CAC) was carried out by reading the MIC levels^12,14^.

Phenotypic interpretation of chromosomal quinolone-resistance mechanisms was based on modification of the method by Kmet and Kmetova^15^. High-level resistance MIC for CIP (≥ 4 mg/L) and ENR (≥ 16 mg/L) involved three mutations in QRDR (*gyrA* and *parC*).

### Genes of antibiotic resistance

ESBL genes for cefotaximases CTX-M^16^, plasmid ampicillinase/cephamycinase CIT^17^, plasmid quinolone resistance genes: *oqxA, oqxB*^18^, *qepA*^19^, *qnrA, qnrB, qnrS* and *aac(6′)IbCr*^20^, integrase 1 (*Int1*)^21^, *Tn3*—transposon^22^ were determined by PCR. DNA sequencing of the PCR products from cefotaximases (CTX-M1) and ampicilinases with primers CIT (CMY-2) was carried out. The O25b-ST131 clone was also detected by PCR^23^.

### Virulence factors

Screening of *E. coli* isolates for ExPEC (Extra intestinal Pathogenic *E. coli*) virulence genes was carried out by PCR amplification of the following: *iutA*—ferric aerobactin receptor; *cvaC*—colicin V and *kpsII*—capsular polysialic acid virulence factor^24^; *iss*—increased serum survival^25^; *tsh*—temperature sensitive haemaglutinin^26^; *papC*—P fimbrial adhesin^27^; and *ibeA*—invasive factor of *E. coli* strains responsible for neonatal meningitis in humans^28^.

### Ethics approval and consent to participate

This study does not qualify for review by the University of veterinary medicine and pharmacy Ethics Board.

## Results

### Antimicrobial susceptibility profiles

**Figure 1:**
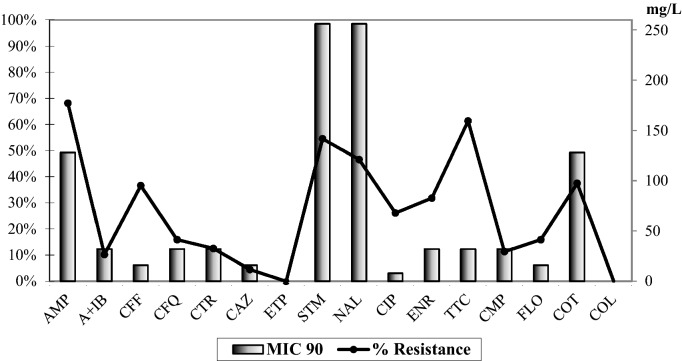
Antibiotic resistance and MIC90 of 88 strains of *E. coli isolated* from rendering plant.

A modified microdilution method with the VetMIC panel was used to detected antimicrobial resistance in 88 *E. coli* strains (Fig. 1).

The highest incidence of beta-lactam resistance was observed for ampicillin (68%), followed by cephalosporins—veterinary ceftiofur (36% and MIC90 16 mg/L) and cefquinome (15.9% and MIC90 32 mg/L), ceftriaxone (12.5% and MIC90 32 mg/L) and ceftazidime (4.5% and MIC90 16 mg/L). Were detected also high resistance to streptomycin (54.5%), tetracyclin (61.4%), florfenicol (15.9%), chloramphenicol (11.39%) and cotrimoxazol (37.5%). All strains were susceptible to ertapenem with MIC 90 0.06 mg/L and colistin. A ratio of ceftazidime MIC to ceftazidime-clavulanic acid MIC greater than 8 indicates the presence of ESBL. From among all 88 investigated *E. coli* strains the ESBL phenotype was confirmed in 18 strains (20.4%). Three phenotypic confirmed ESBL strains from 18 strains which did not present CTX-M or CMY-2 genes are not in the Table 1. However, CMY-2 is related to AmpC.

**Table 1:** Genotyping and antibiotic resistance in 15 selected *E. coli* isolated from rendering plant.

No	ESBL/AmpC	Mobilome	Virulence	Antibiotic resistance	ST131
1	CTX-M1, CMY-2	Int1, Tn3	*cvaC, iutA*	CTR,TTC, STM, FLO, COT	
111	CTX-M1, CMY-2	Int1, Tn3	*cvaC, iutA*	CTR, TTC, STM, FLO, COT	
2	CMY-2	Int1, Tn3	*cvaC, iutA*	ENR	ST131
3	CMY-2	Int1, Tn3	*iutA*	TTC, ENR,COT	ST131
51	CMY-2	Int1, Tn3	*iutA*	TTC, STM, ENR, COT	ST131
21	CMY-2	Int1, Tn3	*iss, ibeA, kps, papC*	CTR, TTC, STM, ENR, COT	
71	CMY-2	Int1, Tn3	*ND*	TTC, FLO	
O3	CMY-2	Int1	*iss*	TTC, STM, ENR	
P1	CMY-2	Int1	*iss, papC*	STM, GEN, ENR,CMP	
K1	CMY-2	Tn3	*iss*	TTC, ENR,COT	
P2		Tn3	*papC*	TTC, ENR, FLO, COT	
O7		Tn3	*papC*	CTR, TTC, STM, NAL	
C51		Int1	*iss, papC,*	CTR, TTC, ENR, CMP, COT	
4C		Int1	*iss, cvaC, iutA, papC*	CTR, TTC, STM,ENR,CMP	
C52		Int1	*iss, iutA, tsh*	CTR, TTC, STM, ENR, CMP, COT	

*iutA* ferric aerobactin receptor; *cvaC* colicin V; *kps* capsular polysialic acid virulence factor; *iss* increased serum survival; *tsh* temperature sensitive haemaglutinin; *papC* P fimbrial adhesin; *ibeA* invasive factor of *E. coli*; *ND* not defined.

Resistance to enrofloxacin was detected in 31.8% (MIC90 32 mg/L) of strains, to ciprofloxacin in 26.1% (MIC90 8.0 mg/L) and to nalidixic acid in 46.5% (MIC90 256 mg/L) of strains. Plasmid-mediated quinolone resistance (*qnr*S) and eflux genes *oqxA, oqxB* and genes *qepA, gnrA, qnrB, qnrS, aac(6′)IbCr* were not detected. High levels of MIC 90 for enrofloxacin and for ciprofloxacin indicate the presence of chromosomal fluoroquinolone resistance^15^.

Multidrug resistance was defined as resistance to at least three non-related antibiotics. Multidrug resistant *E. coli* isolated from rendering plant occurred in 15 strains of *E. coli* (17%), which were selected for PCR analysis (with the exception of *E. coli* strain No. 2).

The strains with the most interesting combination of properties were selected in the Table 1.

### PCR detection of integrons, gene casset, virulence factors, CTX-M and CMY-2

We confirmed an integron-mediated antibiotic resistance in the selected isolates. Class 1 integron cassettes were confirmed in 12 *E. coli* strains, of them the transposons Tn3 gene and six virulence genes *cvaC, iutA, iss, ibeA, kps, papC* were detected in 7 isolates.

The genes *cvaC, iutA, iss, papC,* were the most frequently detected virulence genes detected in *E. coli* strains. Genes of CTX-M1, CMY-2, integrase 1, transposons Tn3, *cvaC* and *iutA* were detected in two strains of *E. coli*.

Three multidrug-resistant clones O25b-ST131 with CMY-2 and enrofloxacin resistance with Int1, Tn 3, *cvaC* and *iutA* were detected.

## Discussion

Critical control points in rendering plants with regard to high level contamination, bioaerosol production and the risk for the environment involve unloading of the raw material and wastewater treatment. In the unloading section of the rendering plant, the raw material is dumped from collecting containers of the transport vehicles to destructors. This is associated with potential aerosolisation of liquids, such as blood, intestinal contents and similar.

Very similar drugs (beta lactams, penicillin, ampicillin, cloxacillin, tetracyclines, sulphonamides and potentiated sulphonamides, cephalosporins, and fluoroquinolones) have been used in both human medicine and agriculture production^29^.

Antimicrobials used in poultry production have the potential to accumulate in poultry feathers and during the rendering process are not completely destroyed. Poultry feathers can be recycled to a feather meal and used as a fertilizer and animal feed, thereby providing a potential pathway for re-entry of drugs into the human food supply^30^.

Hofacre et al.^31^ found that a high percentage of feed samples for poultry containing meat and bone meal from rendering plant were contaminated by bacteria resistant to amoxicillin, ampicillin, cephalothin or clavulanic acid. Some samples contained bacteria resistant to kanamycin, trimethoprim/sulfamethoxazole or ciprofloxacin. The presence of mobile genetic elements mediate multi-drug resistance was proved in many of the isolated bacteria.

Higher than 30% prevalence among the 3rd-generation cephalosporin-resistant *E. coli* was detected mostly in poultry production^32^.

In our study we observed the highest incidence of beta-lactams resistance to ampicillin (68%), followed by cephalosporins—veterinary ceftiofur (36%), cefquinom (15.9%) and ceftriaxone (12.5%) while the beta-lactam resistance to ceftazidime was detected only in 4.5% of all strains.

ESBLs and AmpC beta-lactamases are usually responsible for the mediation of resistance to 3rd-generation cephalosporins in *E. coli*^33^.

Similar ESBL phenotypes with high level fluoroquinolones resistance in animal *E. coli* isolated from a Slovak poultry slaughterhouse was described by Gregova et al*.*^10^. Aggregated European Community data for *E. coli* isolates from broilers showed that over 50% of isolates were resistant to ciprofloxacin^34^. Moreover, the fluoroquinolones-resistant *E. coli* typically exhibited clinically significant elevations in MIC values^35^. *E. coli* isolates resistant to fluoroquinolones are often resistant to other antibiotic groups and genes of virulence^36^.

The CMY-2-producing *E. coli* O25b-ST131 represent a clonal lineage that differs from the CTX-M-15-producing ST131-O25b cluster. ST131-O25b strains with the presence of ESBL-type CTX-M-15 and resistance to fluoroquinolones have been reported worldwide. They are frequently a cause of infections, particularly of the urinary tract of humans. In human patients in Europe, approximately 1% of the 3rd generation cephalosporin-resistant *E. coli* produce CMY-2. However, recent studies in Asia showed higher rates and an increasing trend among the 3rd-generation cephalosporin-resistant *E. coli* isolates has been reported^33^.

Recently, ten multi-resistant strains of *E. coli* that harboured CMY-2 were observed with increasing tendency in the European livestock production. However, ST131 isolates with CMY-2 production have been reported rarely^37,38^.

We also detected multidrug-resistant clones O25b-ST131 with CMY-2 and enrofloxacin resistance with Int1, Tn 3, *cvaC* and *iutA*.

CMY-2-producing *E. coli* isolates were also detected in products from meat, livestock animals and human patients. The predominant way of transmission of *bla*_CMY-2_ genes between animals and humans is the horizontal transfer of temporarily stable *bla*_CMY-2_-carrying IncK2 and IncI1 plasmids^33^. This suggests a zoonotic potential of the *bla*_CMY-2_ genes and their transmission by horizontal transfer and clonal spread along the food production chain^33,37^.

We also detected resistant strains (CTR, TTC, STM, FLO, COT) with genes, such as bla_CTX-M-1_, bla_CMY-2_ together with virulence factors *cvaC, iutA* and mobile elements (Int1, Tn3).

Extraintestinal virulence genes encoding adhesins, iron capture systems, toxins, and protectins have been correlated with successful colonization of gut in humans and animals^39,40^.

Our study of *E. coli* strains from wastewater showed that virulence genes *cvaC, iutA, iss, papC* were the most frequently detected in them*.* In some *E. coli* samples, we detected genes *kps, tsh, papC, ibeA.*

Similarly, examination of meat from healthy broilers from Slovakia conducted by Drugdova et al.^41^ showed presence of antimicrobial-resistant *E. coli* strains with virulence factors (most frequently *iutA, iss, cvaC, tsh* and *papC*) related to avian pathogenic or human uropathogenic *E. coli.*

The study conducted in Canada^42^ revealed high prevalence of many virulence genes (*ompT, traT, uidA, vat, hemF, iss* and *cvaC),* including the genes responsible for adhesion, *fimH* and *kps*MT KII, in ExPEC isolates from frozen poultry meat.

Chicken meat and eggshells also harbour *E. coli* strains containing genes of virulence *papA*, *papC*, *sfa*, *foc, afa*, *dra*, *kpsM* II and *iutA*^43^.

Bok et al.^44^ observed that virulence genes (*fimH, papAH, iutA, iroN, ompT, traT, and iss*) were more frequently identified in isolates from piglets than from sows. *E. coli* from piglets constituted a substantial reservoir of extraintestinal virulence genes and could increase the potential risk of extraintestinal infections. According this study^44^ the mobile genetic elements transmitted via horizontal gene transfer play an important role in the evolution of *E. coli* resistance. Most ExPEC virulence genes are clustered together on mobile genetic elements, usually on pathogenicity islands (PAI) or virulence plasmids, exhibiting a unique organization.

Cunha et al.^45^ characterized APEC strains from different poultry farms in Brazil, that harboured a number of virulence factors such as *sfa*, *usp* (100% each), *pap* (85%), *kps*MTII (66%), *hly*A (52%), *cnf*1 (22%), *ibe*A (4%), *iss* (37%), *tsh, omp*T, and *hly*F (8% each), and *cvi/cva* (0%).

Ten out of 13 tetracycline resistant strains carried the Int1 gene, and 6 of them the *iutA* gene. Five from among 8 streptomycin-resistant strains carried *iutA* and Int1 genes, which indicate a horizontal transfer of resistant genes between bacteria. The high number of isolates resistant to streptomycin, tetracycline and cotrimoxazol can be spread by same mobile genetic elements.

## Conclusion

In conclusion, the present investigations illustrated the current state of antibiotic resistance of *E. coli* strains in the investigated rendering plant. We detected the presence of *E. coli* with CTX-M, cephamycinase CMY-2 genes and high level of chromosomal resistance to fluoroquinolones. Furthermore, we found three CMY-2 producing *E. coli* O25b-ST131, resistant to a high level of enrofloxacin with *cvaC* and *iutA* virulence factors. The CMY-2 producing *E. coli* isolates have a zoonotic potential and pose a serious health risk.

Considering that rendering plant is an important source of resistant bacteria, our data highlight the importance of adequate protection of the working personnel and observation of strict hygiene measures at operation premises.
